# Ketamine—New Possibilities in the Treatment of Depression: A Narrative Review

**DOI:** 10.3390/life11111186

**Published:** 2021-11-05

**Authors:** Mateusz Kowalczyk, Edward Kowalczyk, Paweł Kwiatkowski, Łukasz Łopusiewicz, Monika Sienkiewicz, Monika Talarowska

**Affiliations:** 1 Babinski Memorial Hospital, Aleksandrowska St. 159, 91-229 Lodz, Poland; mateuszjerzykowalczyk@gmail.com; 2 Department of Pharmacology and Toxicology, Medical University of Lodz, Żeligowskiego St. 7/9, 90-752 Lodz, Poland; edward.kowalczyk@umed.lodz.pl; 3 Department of Diagnostic Immunology, Pomeranian Medical University in Szczecin, Powstańców Wielkopolskich 72, 70-111 Szczecin, Poland; pawel.kwiatkowski@pum.edu.pl; 4 Center of Bioimmobilisation and Innovative Packaging Materials, Faculty of Food Sciences and Fisheries, West Pomeranian University of Technology Szczecin, Janickiego 35, 71-270 Szczecin, Poland; lukasz.lopusiewicz@zut.edu.pl; 5 Department of Pharmaceutical Microbiology and Microbiological Diagnostic, Medical University of Lodz, Muszyńskiego St. 1, 90-151 Lodz, Poland; 6 Department of Clinical Psychology and Psychopathology, Institute of Psychology, University of Lodz, Smugowa St. 10/12, 91-433 Lodz, Poland; monika.talarowska@now.uni.lodz.pl

**Keywords:** ketamine, depression, anxiety, NMDA receptor, neurodegeneration, GABA, COVID-19 pandemic

## Abstract

The SARS-CoV-2 coronavirus epidemic has led to an increase in the number of people with depression. Symptoms related to the mental sphere (mainly depression and anxiety) may be experienced by one third of the worldwide population. This entails the need for the effective and rapid treatment of depressive episodes. An effective drug seems to be s-ketamine, which was accepted in March 2019 by the Food and Drug Administration (FDA) for the treatment of drug-resistant depression. This drug provides a quick antidepressant effect with maximum effectiveness achieved after 24 h. It also appears to reduce the occurrence of suicidal thoughts. However, research into undesirable effects, especially in groups of people susceptible to psychotic episodes or those who use alcohol or psychoactive substances, is necessary.

## 1. Introduction

As of 1 May 2021, there were 153 million confirmed COVID-19 cases, and 3.2 million related deaths have been reported by the WHO. The regions of the Americas and Europe have been the most affected (almost half (48%) of all reported COVID-19-associated deaths occurring in the Americas, and one third (34%) in Europe) [1]. According to the World Health Organization, before the COVID-19 pandemic, approximately 350 million people worldwide were suffering from depression [2]. The worldwide distribution of so-called *major depression* (MD) or *major depressive disorders* (MDD) was estimated from 3% in Japan to 16.9% in the USA; in most other countries the prevalence ranged between 8% and 12% [3,4].

The SARS-CoV-2 coronavirus epidemic has produced changes in these statistics for several months. According to the available data, symptoms related to the mental sphere (mainly depression and anxiety symptoms) may affect one third of the general population [5]. According to Santomauro et al., there has been an increase of 27.6% per 100,000 population cases of major depressive disorder globally due to the COVID-19 pandemic, with increases in North Africa and the Middle East of 37.2%, in Latin America and the Caribbean of 37.8%, and in Central Europe, Eastern Europe, and Central Asia of 29.4%. The authors also estimated an increase of 25.6% per 100,000 population cases of anxiety disorders globally, mainly in South Asia, where an increase of 35.1% was noted, but also in North Africa and the Middle East, where there has been an increase of 32.4%, in Latin America and the Caribbean, with an increase of 31.7%, and in Central Europe, Eastern Europe, and Central Asia, with an increase of 30.0% [6]. In a broader perspective, depressive disorders in the time of the COVID-19 pandemic have many negative consequences, both medical and sociological, and significantly reduce both quality of life and the adaptability of the individual [7].

Depression is a complex and heterogeneous disease, and its etiopathogenesis involves many factors at different psychological, biological, genetic and social levels [8]. Despite the great medical and social importance of MD, there is no clear concept behind its causes and the mechanism of its development. Several theories explaining the onset of depression have been proposed and confirmed by biochemical, immunological and physiological studies. In parallel to the well-known theories of depression (“monoamine”, “cytokine”, “stress”—the hypothalamic–pituitary–adrenal (HPA) axis), other theories, including altered brain neural plasticity, neurogenesis and circadian desynchronosis (chronobiological model), have been proposed to explain the onset of MD symptoms [9,10].

A detailed understanding of the mechanisms underlying depression appears to be essential to provide an appropriate therapeutic approach in order to alleviate or arrest the disease. Guidelines for treating depression suggest selective serotonin reuptake inhibitors (SSRIs) and serotonin norepinephrine reuptake inhibitors (SNRIs) among other drugs, including agomelatine, bupropion, mirtazapine and vortioxetine as first-line drugs [11,12]. However, about one-third of depressed patients treated report little or no symptomatic improvement. This phenomenon often leads to discontinuation of therapy and deterioration in the quality of patients’ lives and increases the risk of successful suicide attempts [13].

Over the past few years, ketamine has become more popular as a potential new drug in the pharmacotherapy of MD [14,15]. It has been successfully used as a general fast-acting anesthetic for a long time. Ketamine is a derivative of phencyclidine, a substance belonging to the group of psychodysleptic agents, also known as hallucinogens [16].

In March 2019, the Food and Drug Administration (FDA) approved the use of intranasal s-ketamine for the treatment of drug-resistant depression [17]. There have been more and more reports indicating the effectiveness of this substance in the therapy of so-called treatment-resistant depression (TRD) [18]. Bartoli et al. report that ketamine significantly reduces the frequency of suicidal thoughts in depressive patients [19]. McIntyre et al. state that intravenous ketamine is an effective antidepressant in people with the above-mentioned drug-resistant depression [20]. Will the N-methyl-D-aspartate (NMDA) receptor potentially be a new treatment target for depression [21]?

## 2. Methodology

An exhaustive review of the literature published up to October 2021 in PubMed (U.S. National Library of Medicine) was performed. Our search strategy combined the following terms: ketamine, depression, NMDA receptor, neurodegeneration, GABA. The full articles were downloaded, as well as books on the treatment of depression, to find possible additional related publications.

## 3. N-Methyl-D-Aspartate (NMDA) Receptor

The stimulating effect of amino acids on the activity of neurons was first described by Curtis in 1959 [22]. He showed a depolarizing effect of glutamic acid (GLU) on rat spinal neurons. Twenty consecutive years have been spent on research proving that GLU is one of the most important excitatory neurotransmitters in the central nervous system (CNS) [23], and that glutamate receptors are ubiquitous in the brain and spinal cord. There are three families of ionotropic glutamate receptors: N-methyl-D-aspartate (NMDA)—the agonist is NMDA; α-amino-3-hydroxy-5-methyl-4-isoxazole propionate (AMPA)—the agonist is AMPA; and kainic—stimulated by kainic acid. There are many binding points of the so-called competitive antagonists (the same site where GLU and NMDA bind) within the NMDA receptor [24,25].

**Figure 1:**
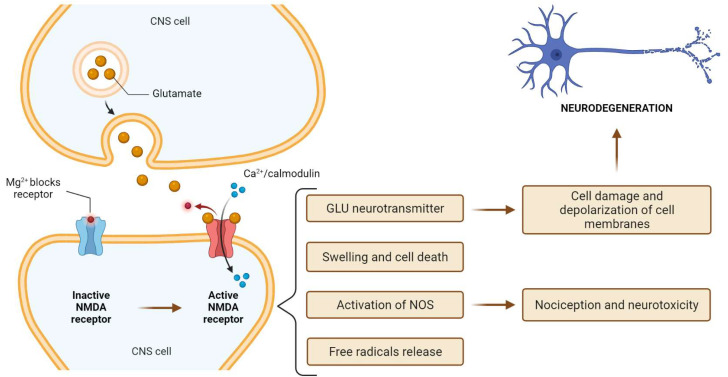
The effects of NMDA receptor activation. Created with BioRender.com (accessed on 1 September 2021).

At rest, the NMDA receptor is inactive due to the voltage-dependent blocking of the channel pores by magnesium ions. Unlike AMPA, NMDA receptors are non-selective for cations such as Ca^2+^, Na^2+^ and K^2 +^ ions. Ca^2+^ influx is crucial for NMDA receptor induction. The influx of calcium into the cell activates calcium-dependent enzymes, stimulates the formation of free radicals and leads to swelling and cell death. It also leads to the release of a large amount of GLU, which is found in significant amounts in CNS cells. Glutamate outside the cell can depolarize cell membranes, damage subsequent cells and is responsible for the spread of the neurodegeneration process. This glutamate loop can increase the extent of damage after trauma, ischemia and encephalitis [26]. Ca^2+^/calmodulin-mediated activation of the NMDA receptor also leads to the activation of nitrogen oxygen synthetase (NO), which plays a key role in nociception and neurotoxicity. The results of NMDA receptor activation are shown in Figure 1.

Already in the 1950s, Lucas and Newhouse [27] presented the possible toxic effects of GLU on cells. However, the author of the concept of neurotoxicity of excitatory amino acids in the brain is Olney [28], who described the mechanism of excessive stimulation of NMDA receptors by GLU in the pathogenesis of various forms of nerve cell damage. He also drew attention to the role of NMDA receptors and the possibility of modulating the glutamatergic system function through antagonists of these receptors.

The functioning of the glutamatergic system determines the proper functioning of cognitive processes, memory and learning. The results of many studies indicate a relationship between the dysfunction of the glutamatergic system and neuroplasticity disorders and neurodegenerative processes in the course of Alzheimer′s or Huntington′s disease [29]. NMDA receptors are involved in global and focal ischemia and in various neurological diseases [30].

Scientific research confirms the involvement of the glutamatergic system in the processes of mood regulation. People suffering from depression showed a decreased level of this neuromediator in the cingulate cortex, with elevated levels in the occipital cortex. [31]. Dysfunction of AMPA receptors (including the decreased expression of mGlu3 subunits) in the prefrontal cortex and the increased expression of NMDA receptors were reported in patients suffering from affective disorders [32].

In addition, the glutamatergic system is involved in neuroplasticity processes, such as the formation and proper functioning of synapses, axon and dendrite cytoskeleton and in the maintenance of the level of neuromediators. It is possibly involved in all CNS functions [33,34,35]. The influence on the glutamatergic system is therefore the basis of intervention in many brain dysfunctions.

The renaissance in anesthetic ketamine as the first non-competitive NMDA receptor antagonist is currently noted. Non-competitive NMDA receptor antagonists act at a site other than the agonist recognition site by blocking the NMDA receptor in the channel in a usage-dependent manner (i.e., the channel must first be opened by the agonist in order to be bound to the antagonist).

New research also indicates other potential mechanisms of NMDA receptor antagonist action protecting against the progression of neurodegeneration. According to this work, for instance, amantadine may exhibit a double protective mechanism, completely independent of blocking the NMDA receptor and consisting in both the inhibition of microglia stimulation (and thus the inflammatory process) and the astroglial stimulation (thus the production of trophic factor (GDNF—glial derived neurotrophic factor)). Today, many researchers consider both of these phenomena, the microglia stimulation and reduction in trophic factors, as crucial in the processes of neurodegeneration and depression [36,37,38].

## 4. Ketamine Characteristics

Ketamine is a synthetic, non-barbiturate anesthetic synthesized by Calvin Stevens of the Parke-Davis Pharmaceutical Company in 1962 (Ann Arbor, MI, USA), which was looking for an alternative to the potent hallucinogenic agent phencyclidine [39,40].

Due to its rapid onset and short duration of action, with only minor cardio-respiratory depression compared to other general anesthetics, and possible use in maintaining anesthesia via inhalation, ketamine is the drug of choice for short-term surgery, especially in children, and is also used in veterinary medicine [41,42]. Ketamine produces dissociative anesthesia (i.e., a sense of separation from the body and surroundings) [40,43]. Under dissociative anesthesia, the patient remains awake and seems to be conscious (i.e., the eyes may be open with nystagmus present), but shows no response to surgical pain. He/she is in complete analgesia and total amnesia, with reflex protection in the airways (intubation is not necessary), spontaneous breathing and cardiovascular stability (i.e., blood pressure and heart rate do not drop and may even increase slightly) [44]. The dissociative state appears to result from functional dissociation—inhibition of the thalamocortical pathways and stimulation of the limbic areas of the brain [45]. These neural systems help maintain the neural connections required for consciousness. Ketamine is also proposed as an effective pain reliever and a therapeutic agent in the treatment of alcoholism [46], heroin addiction [47] and anorexia [48]. It is recommended in the treatment of depression due to its long-lasting effects and quick onset of action (within four hours after administration) [49,50,51,52].

In 2000, Berman et al. demonstrated that a dose (0.5 mg/kg) of ketamine exerts a rapid and long-lasting antidepressant effect in patients with MDD [53]. Zarate et al. confirmed the rapid and long-lasting antidepressant effect of ketamine in patients with refractory MDD and found that ketamine reduces the tendency to have suicidal thoughts in patients with MDD [54]. Although ketamine has a strong antidepressant effect, its side effects may limit widespread use [55,56,57].

Ketamine (Ki = 0.53 µM for NMDAR) racemic mixture with different proportions of (R)-ketamine and (S)-ketamine.

Binder affinity (S) of ketamine (Ki = 0.30 μM) to NMDAR is about four times stronger than (R)-ketamine (Ki = 1.4 μM).

The anesthetic effect of (S)-ketamine is approximately three to four times stronger, but the psychomimetic side effects are greater than those of (R)-ketamine. Studies showed that (R)-ketamine has a stronger antidepressant effect than (S)-ketamine. Recent studies showed that the order of antidepressant effects after intranasal administration is: (R)-ketamine > (R, S)-ketamine > (S)-ketamine; and that the order of adverse effects in rodents is (S)-ketamine > (R, S)-ketamine > (R)-ketamine. As was mentioned in the introduction, on 5 March, 2019, the US FDA approved (S)-ketamine nasal spray (Spravato ™) for cases of treatment-resistant depression [58]. A clinical trial of (R)-ketamine and (2R, 6R)-HNK in humans is currently underway.

## 5. Ketamine—The Receptor and Non-Receptor Mechanism of Action

Ketamine has been known for many years as an antagonist of glutamate receptors [21]. Like phencyclidine, ketamine blocks the NMDA receptor in a non-competitive manner. It blocks the open channel and reduces the average channel open time. Ketamine also reduces the frequency of channel openings [59]. NMDA receptor antagonism is responsible for the specific properties of ketamine, such as amnesia and its psychosensory, analgesic and neuroprotective effects. Ketamine also has glutamate-independent effects. It interacts with many receptors, such as opioid, monoaminergic, cholinergic, nicotinic and muscarinic receptors. It potentiates the inhibitory effects of GABA (GABA-A complex) [60]. Ketamine binds to the mu, delta and kappa opioid receptors. The affinity of S(+)-ketamine for opioid receptors is two to three times higher than that of the R (−) isomer. The effect of ketamine on opioid receptors is not antagonized by naloxone [61].

Undoubtedly, its action on the monoaminergic system is important. Ketamine causes a hyperadrenergic state (i.e., stimulates noradrenergic neurons and inhibits the uptake of catecholamines, increasing the release of norepinephrine, dopamine and serotonin).

Inhibition of norepinephrine uptake is stereospecific, the R (−) isomer only inhibits its uptake by neurons, while the S (+) isomer also inhibits extra neuron uptake. There is a prolonged synaptic action leading to an increased transfer of norepinephrine into the blood circulation [59]. Alpha-2 agonists are able to reduce this hyperadrenergic state and also the psychological phenomena caused by ketamine [62]. Due to its interaction with the serotonin transporter [63], ketamine also inhibits the uptake of dopamine and serotonin [64]. Some of the effects of ketamine include the purinergic system, such as urinary toxicity [65].

Ketamine interacts with sodium channels. It binds to the same site inside sodium channels as local anesthetics [66]. It is also effective as a topical pain reliever [67]. Moreover, ketamine inhibits neuronal potassium channels [68]. This mechanism may explain some of the neuroprotective properties of S (+) isomers.

**Figure 2:**
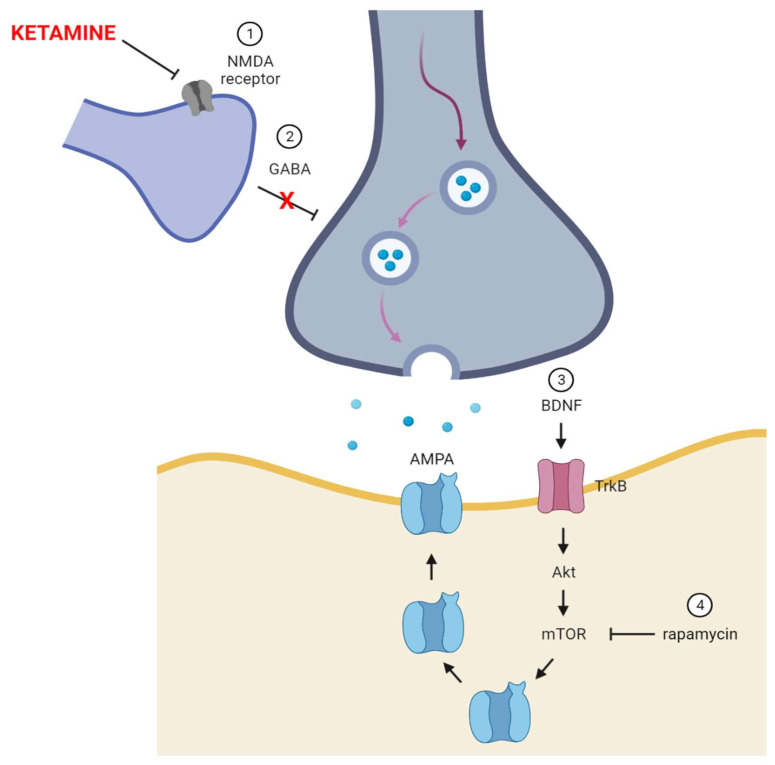
Mechanism of the antidepressant action of ketamine. Created with BioRender.com (accessed on 1 September 2021).

Several studies suggest that ketamine, through glutamate and/or neurotrophic receptors, stimulates the mammalian target of rapamycin (mTOR) pathway in the prefrontal cortex (PFC) [51,69,70]. mTOR is a threonine–serine protein kinase involved in cell proliferation, mortality, survival and protein synthesis [71], including the neurotrophic growth factor (BDNF) that binds to the neurotrophin receptor (TrkB). Studies showed that chronic ketamine treatment was able to counteract the decline in BDNF protein in the hippocampus and nucleus accumbens [72]. The process of antidepressant action of ketamine was shown in Figure 2.

Heise et al. demonstrated that ketamine increases the BDNF expression and exerts antidepressant effects in experimental animals, but not in knockout animals of the eukaryotic elongation factor 2eEF2K kinase [73]. eEF2K is a kinase important for the regulation of protein translation elongation [74]. Liu and Dumni believe that eEF2K can play a role in processes such as learning and memory, and in depression [75]. Ketamine activation of mTOR can be suppressed by rapamycin, a specific mTOR inhibitor. Rapamycin blocks ketamine-induced synaptogenesis and abolishes the antidepressant effect of ketamine [51].

## 6. Ketamine Toxicity

Being an analog of phencyclidine, ketamine is ten times less potent and causes less severe dysphoria and hallucinations. Unfortunately, the psychomimetic effects made ketamine a popular party/club drug. It produces euphoric and dissociative effects at low doses and immobilizing and hallucinogenic effects at high doses.

The 2015 World Drug Report classified ketamine as a worldwide recreational drug. However, ketamine abuse is relatively minor, and phencyclidine derivatives account for only 1% of the “new psychoactive substances”. This type of ketamine use often occurs in conjunction with other substances, including alcohol, amphetamines, MDMA, cocaine and caffeine. In the United States, the abuse of ketamine has increased since the 1980s. However, compared to the sharp increase in opioid abuse and the illegal abuse of cannabis, the abuse of ketamine is relatively small. Ketamine use is becoming increasingly popular as a recreational drug in Southeast Asian countries, such as Taiwan, Malaysia, and China [76].

Currently, there is little information on the toxicokinetics of ketamine in the human population. Gable estimated the mean lethal dose to be 600 mg/kg, or 4.2 g for a 70 kg human, on average [77].

The symptoms of ketamine overdose are similar to those of phencyclidine overdose, although the effects of ketamine wear off more quickly. Physical signs and symptoms depend on the dose and interactions with other compounds taken at the same time. General symptoms, including sedation and disturbed consciousness, may occur when using ketamine. In addition, horizontal, vertical or rotational nystagmus, pupil dilation and excessive salivation are observed. Moreover, cardiovascular symptoms, such as hypertension, tachycardia, palpitations, arrhythmias and chest pain, have been noted. Abdominal pain, tenderness, nausea and vomiting may occur. The neurological symptoms include an altered mental state (confusion), paranoia, dysphoria, restlessness, confusion, slurred speech, dizziness, ataxia, dysarthria, trismus and muscle stiffness. Less pain sensation affects the possibility of injuries occurring [78,79,80].

Usually, in patients with symptoms of ketamine poisoning, observation and, if necessary, symptomatic treatment are sufficient. The effects of ketamine poisoning usually last from 15 min to several hours depending on the dose, route of administration, metabolic capacity and intrinsic sensitivity to the effects of the drug [81].

Patients who are asymptomatic but report recent ketamine use should be followed up for six hours. Patients who experience mild symptoms of intoxication should be monitored for 1–2 h after the last symptoms have subsided. Monitoring includes observation of respiration and circulation because ketamine has the potential to cause cardiopulmonary disorders, especially when it is taken in combination with other medications.

There are no medications approved by the US Food and Drug Administration to treat ketamine overdose, but medications can help manage agitation and psychosis. Benzodiazepines, such as lorazepam and diazepam can relieve agitation, psychomimetic effects, hypertension, hyperthermia and seizures. Lorazepam is usually given at a dose of 2 to 4 mg intravenously or intramuscularly, and diazepam is usually given intravenously at a dose of 5 to 10 mg. Butyrophenones, including haloperidol, have been used to treat psychotic and agitation episodes [81].

In hyperthermia, sedation is used, and when it is ineffective, cold compresses are applied. Drugs that help relieve symptoms include alpha-2 agonists, such as clonidine. Clonidine can treat or prevent the psychomimetic side effects of ketamine, increase hemodynamic stability by lowering blood pressure and provide synergism with the analgesic effect of ketamine [82,83]. Clonidine is usually administered at a dose of 2.5–5 mg/kg by oral administration. Atropine or glycopyrrolate can prevent and treat hypersalivation associated with ketamine use, while physostigmine can treat nystagmus and blurred vision. In turn, administration of crystalloids may improve hydration. Hemoperfusion and dialysis are usually unsuccessful in ketamine intoxication due to the large volume of ketamine distribution [80].

## 7. Discussion

Studies seem to confirm the effectiveness of using s-ketamine in MDD. In the long-term studies, the majority of patients responded to the treatment with 84 mg, and about one third of patients responded to 56 mg, given weekly or every other week. The results also suggest that s-ketamine reduces the risk of suicide [84,85,86,87]. The adverse dissociative symptoms occurred within about 30 to 40 min following its administration and subsided after two hours. The increase in blood pressure lasting 10 to 40 min was recorded, which usually subsided two hours after the drug was administered. Other side effects included dizziness, headache, dysgeusia, sedation and nausea. Only dizziness and nausea were dose dependent. The studies were carried out in groups of patients taking s-ketamine in a dose of 14 mg, 28 mg, 56 mg and 84 mg [88,89]. The most commonly reported side effects of s-ketamine in people with depression are dizziness (67%), nausea (37.5%), disturbance in attention (29.2%) and fatigue (29.2%) [85].

## 8. Conclusions

Ketamine is the first drug found to exert an impact on treatment-resistant depression with immediate effect. It also seems to reduce the occurrence of suicidal thoughts and provides a quick antidepressant effect with maximum effectiveness achieved after 24 h. The efficacy and safety of its medium- and long-term use have not yet been well researched. Evidence from previous studies suggests that ketamine significantly reduces the severity of depression. However, extrapolation of these results should be attempted cautiously, as the patients included in the studies have not had a history of psychotic episodes or disorders related to the use of alcohol or psychoactive substances, which is not representative of all depressed patients who may benefit from this therapy.

## Data Availability

PubMed (U.S. National Library of Medicine).
